# Technical and clinical validation of commercial automated volumetric MRI tools for dementia diagnosis—a systematic review

**DOI:** 10.1007/s00234-021-02746-3

**Published:** 2021-09-03

**Authors:** Hugh G. Pemberton, Lara A. M. Zaki, Olivia Goodkin, Ravi K. Das, Rebecca M. E. Steketee, Frederik Barkhof, Meike W. Vernooij

**Affiliations:** 1 Centre for Medical Image Computing (CMIC), Department of Medical Physics and Bioengineering, University College London, London, UK; 2 UCL Queen Square Institute of Neurology, University College London, London, UK; 3 Dementia Research Centre, UCL Queen Square Institute of Neurology, University College London, London, UK; 4 Department of Radiology and Nuclear Medicine, Erasmus MC University Medical Center, Rotterdam, The Netherlands; 5 Clinical, Educational and Health Psychology, University College London, London, UK; 6 Radiology & Nuclear Medicine, VU University Medical Center, Amsterdam, The Netherlands; 7 Department of Epidemiology, Erasmus MC University Medical Center, Rotterdam, The Netherlands

**Keywords:** AI, Quantitative MRI, Neuroradiology, Volumetric, Dementia diagnosis, Atrophy

## Abstract

Developments in neuroradiological MRI analysis offer promise in enhancing objectivity and consistency in dementia diagnosis through the use of quantitative volumetric reporting tools (QReports). Translation into clinical settings should follow a structured framework of development, including technical and clinical validation steps. However, published technical and clinical validation of the available commercial/proprietary tools is not always easy to find and pathways for successful integration into the clinical workflow are varied. The quantitative neuroradiology initiative (QNI) framework highlights six necessary steps for the development, validation and integration of quantitative tools in the clinic. In this paper, we reviewed the published evidence regarding regulatory-approved QReports for use in the memory clinic and to what extent this evidence fulfils the steps of the QNI framework. We summarize unbiased technical details of available products in order to increase the transparency of evidence and present the range of reporting tools on the market. Our intention is to assist neuroradiologists in making informed decisions regarding the adoption of these methods in the clinic. For the 17 products identified, 11 companies have published some form of technical validation on their methods, but only 4 have published clinical validation of their QReports in a dementia population. Upon systematically reviewing the published evidence for regulatory-approved QReports in dementia, we concluded that there is a significant evidence gap in the literature regarding clinical validation, workflow integration and in-use evaluation of these tools in dementia MRI diagnosis.

## Introduction

In the clinical diagnosis of dementia, structural MRI plays a key role in excluding other pathologies, as well as revealing patterns of brain atrophy [1, 2]. These patterns can act as imaging biomarkers to assist nosological diagnosis and differentiation between subtypes of dementia [3]. In clinical neuroradiology, visual assessment of brain atrophy patterns is commonly supported through the use of visual rating scales, such as the global cortical atrophy (GCA) or medial temporal atrophy (MTA) scale [4]. These semi-quantitative measures have shown good diagnostic accuracy to distinguish dementia from normal ageing and can help mediate the subjectivity of visual assessment [5]. However, they are sensitive to the experience and perspective of the clinician and can be limited by their relatively coarse measurement of atrophy and floor and/or ceiling effects [6, 7]. These qualities make it difficult to use such scales to identify subtle volumetric abnormalities in younger patients. Also, sensitivity to abnormalities in prodromal dementia patients is still limited [7]. With the focus on developing prophylactic and disease-modifying treatments for dementia, the need for robust methods of distinguishing between healthy ageing and dementia in its early stages is increasingly important [8].

These needs can potentially be addressed through the implementation of automated quantitative image analysis in the clinic. Volumetry is widely used in the research setting and has been used to effectively index morphological change from a variety of clinical interventions in phased and randomized controlled trials [9–17]. Quantitative volumetric reporting tools (QReports), which automatically quantify an individual patient’s regional brain volumes and compare them to healthy, age-specific reference populations, can potentially help neuroradiologists interpret the severity and distribution of brain atrophy and contextualize their findings by referencing normative brain volumes in healthy populations [18–23]. The limitations of routine visual assessment reveal the area of clinical need in which such tools can be integrated. Quantitative assessment of MRIs can provide more objective imaging biomarkers, contribute to the earlier identification of atrophy [24–26] and might improve the accuracy of radiological diagnosis of Alzheimer’s disease (AD) and other subtypes of dementia [18–23]. However, there remains a large discrepancy between the use of visual rating scales and the availability of QReports in the clinic. In a study of dementia imaging practices in Europe, 81.3% of the 193 centres surveyed reported routine use of the MTA scale, compared to only 5.7% regularly implementing QReports [27]. Respondents identified limited availability and concerns about time and interpretation difficulties as the barriers for use of these tools. Importantly, the survey also recognized the additional obstacles to implementation, including lack of standardization or clinical validation of proprietary tools, and the difficulty translating normative group-level quantitative data to the interpretation of *individual patient data*.

With the surge of commercial QReports for application in dementia clinics, general radiologists and neuroradiologists must decide whether to start implementing these methods in their clinical practice. However, there is a scarcity of evidence regarding the clinical application of QReports, especially relating to the impact on clinical management. It is important to clarify their technical and clinical validity as well as the best practices for responsibly integrating these tools into the existing clinical workflow. To this end, the quantitative neuroradiology initiative (QNI) was developed as a framework for the technical and clinical validation necessary to embed automated image quantification software into the clinical neuroradiology workflow. The QNI framework comprises the following steps: (1) establishing an area of clinical need and identifying the appropriate proven imaging biomarker(s) for the disease in question; (2) developing a method for automated analysis of these biomarkers, by designing an algorithm and compiling reference data; (3) communicating the results via an intuitive and accessible quantitative report; (4) technically and clinically validating the proposed tool pre-use; (5) integrating the developed analysis pipeline into the clinical reporting workflow and (6) performing in-use evaluation [2].

The aim of this review is to increase transparency by assessing the evidence surrounding the use of QReports according to these six steps. Evidence of step 1 has been outlined above; the area of clinical need we are addressing is dementia and the analysis of its associated volumetric biomarkers. Using steps 2–6 of the QNI framework as guidance, we present a systematic search methodology for finding (i) vendors of dementia and MRI-specific QReports that are either *Conformité Européenne* (CE) marked or certified by the Food and Drug Administration (FDA) and (ii) published evidence covering their technical/clinical evaluation and workflow/in-use evaluation. Furthermore, we present an unbiased narrative synthesis of the available evidence regarding the validation of volumetric tools applied in the memory clinic. In doing so, we aim to help neuroradiologists make informed decisions regarding these tools in their clinic.

## Methods

The methods used to find relevant companies and QReports are outlined below. The vendor and product names identified were subsequently used as the search terms for an extensive search of the technical/clinical validation and workflow/in-use evaluation studies in the literature. We have followed Preferred Reporting Items for Systematic Reviews and Meta-Analyses (PRISMA) guidelines [28–30] and our methodology has been registered in with the Prospective Register of Systematic Reviews (PROSPERO): number CRD42021233510.

### Vendor and product search

#### Inclusion and exclusion criteria

The following inclusion criteria for proprietary QReports were used: (i) FDA or CE clearance, i.e. tool meets regulatory standards to be used clinically; (ii) target disorder of dementia/neurodegeneration, specified by companies for use in dementia MRI assessment; (iii) uses automated brain segmentation software (step 2 of the QNI framework); (iv) uses normative reference data for single-subject comparison; (v) MRI-based input and (vi) visualizes volumetry and atrophy-specific results presented in a structured report format (step 3 of the QNI framework).

Our exclusion criteria for proprietary products were (i) research tools that are not currently certified for clinical use via CE or FDA approval; (ii) non-MRI-based tools, e.g. for PET, EEG or CT only; (iii) generates a QReport focusing on results other than volumetry/atrophy, e.g. white matter lesions, vasculature, electrophysiology, tractography, brain tumour analysis or PET/spectroscopy; (iv) lack of normative reference data for single-subject comparison.

### Search methodology: FDA-cleared product identification

#### Key word screening

We used the FDA database search function to download basic information for each approved application (https://www.accessdata.fda.gov/scripts/cdrh/cfdocs/cfPMN/pmn.cfm). A total of 82,003 premarket 510(k) FDA notification clearances dating from 1996-present were downloaded in a text file from https://www.fda.gov/medical-devices/510k-clearances/downloadable-510k-files. By searching within this list using the keywords mentioned below, 828 “medical devices” were established for further review. Please note the words with an * are “wild-cards”, covering relevant suffixes of each word stem, for example “Radiolog*” covers “Radiology”, “Radiologist” and “Radiological”:

- Neuro*
- Brain
- Quant*
- MRI
- Hippocamp*
- Radiolog*
- Atroph*
- Cortical
- Cortex
- Dementia
- Volume
- Alzheimer*
- Memory
- Lobar
- Lobe
- Structur*
- Segment*
- Automat*

#### Eligibility screening

**Fig. 1:**
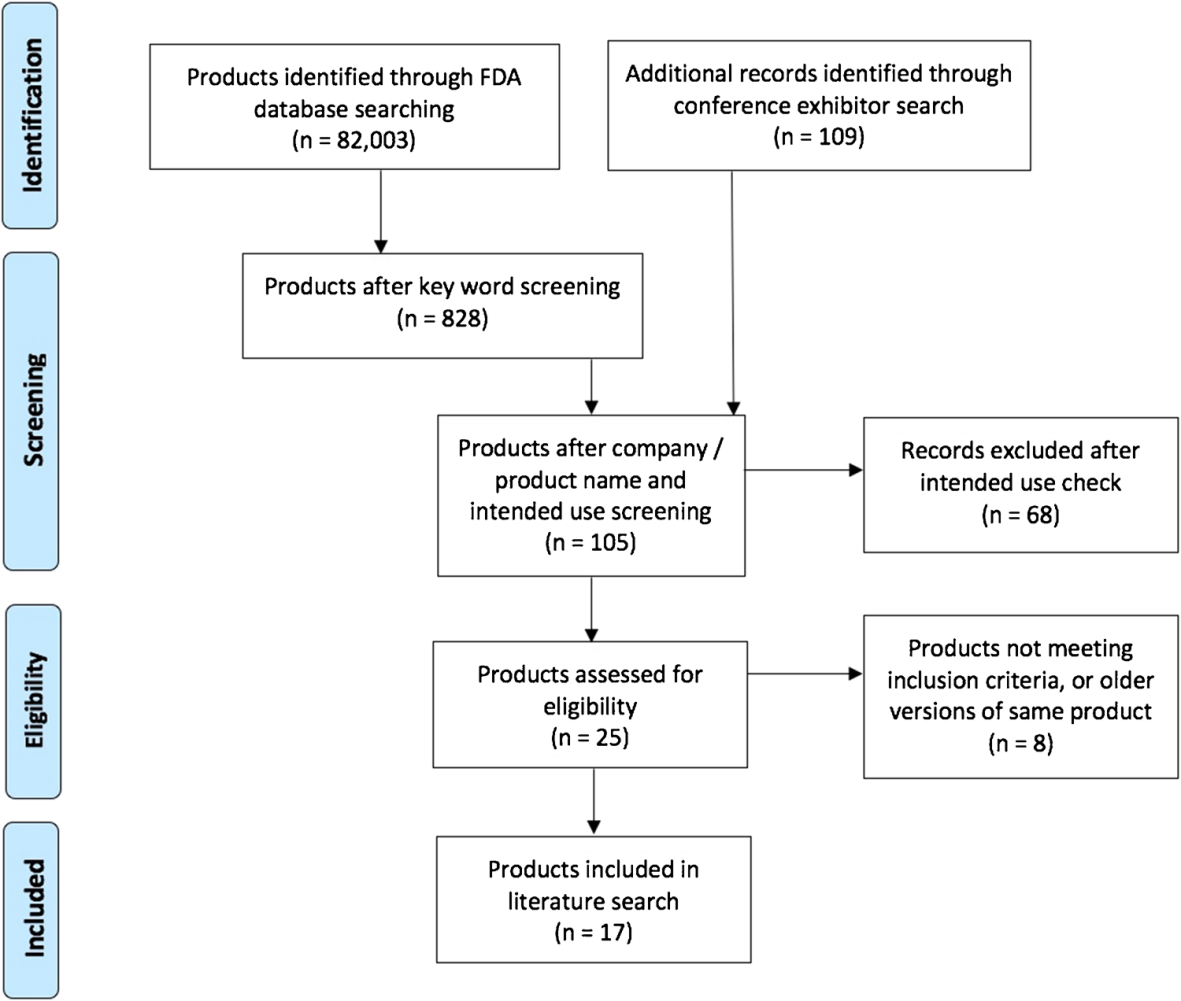
Research flowchart showing a systematic and extensive search for CE marked and FDA cleared QReports. Websites of companies exhibiting at the most recent ISMRM, ESMRMB, RSNA, ECR, ESR AIX, ASNR, SIIM and ESNR were searched, and the website https://grand-challenge.org/aiforradiology/ was cross-checked

After manual checks of company name, date of approval, product name and description, 86 tools were deemed relevant for further examination. Several tools were excluded at this stage if their description mentioned other body parts, for example “wrist array coil”, or were considered hardware. After investigating their intended uses on the FDA application and company website, 28 tools required further checking. After removing older versions of the same software, 16 relevant tools were assessed against our inclusion criteria, after which 9 companies/QReports remained (see Fig. 1 for PRISMA flowchart).

### Search methodology: CE-marked product identification

Unfortunately, there is no freely available and searchable database of CE-marked medical devices yet, although plans are underway to deploy one this year (EUDAMED) [31]. Therefore, the same comprehensive method used by the FDA could not be applied. In lieu of this, detailed review of the websites of companies exhibiting at the most recent relevant medical imaging conferences (ISMRM, ESMRMB, RSNA, ECR, ESR AIX, ASNR, SIIM and ESNR) were used to find CE-marked quantitative tools. The website https://grand-challenge.org/aiforradiology/ was also used to cross-check the results. One hundred and nine companies were identified for further investigation; after checking the information on their websites against our inclusion criteria and following up with direct email contact where necessary, 8 were included.

### Company and product features

Given a large number of companies and wide range of features, one aim of this review is to provide an unbiased repository of technical features and characteristics to help clinicians and researchers select the most appropriate QReports for their individual investigations. After establishing a list of companies that met our inclusion criteria, all vendors were contacted to provide relevant information that was unavailable on their websites. The following features, deemed to be most relevant to clinicians and researchers, were decided in advance and then sought through website research and direct vendor contact:

- CE/FDA approval status
- Date of approval
- Target disorder
- Segmentation/volumetry method
- Lobar and sub-lobar parcellation/volumetry
- Cross-sectional only or also longitudinal analyses available
- Report processing time
- Details of a normative reference population
- Provision of segmentation overlays/atrophy heat maps
- Strategies to account for inter-scanner variability
- Image quality control method
- Report deployment/PACS integration procedure

When all information had been collected, we contacted vendors again for final confirmation of their individual details prior to publication.

### Literature search on technical and clinical validation of identified products

The results of this systematic review are intended to help inform potential users of QReports, assumed to mainly be clinicians. Given the health-related implications of the results and in the interest of reproducibility, the methodology has been registered with the PROSPERO — Registration Number: CRD42021233510. In line with the PRISMA guidelines [28–30], a detailed search was conducted using the identified company and associated QReport names as search terms. Both names were searched in order to cover the full breadth of technical and clinical validation papers in the literature and to cover research conducted pre-branding or product naming. PubMed, Scopus and Ovid Medline “All fields” were accessed (latest search on 15 March 2021) using the search terms below; brackets are used to indicate that a term consisting of multiple words was used as a single search term:

1. (ADM diagnostics) OR (Corinsights MRI)
2. Brainminer OR diadem
3. Brainreader OR neuroreader
4. Combinostics OR cNeuro
5. CorTechs OR NeuroQuant
6. Corticometrics OR THINQ
7. Icometrix OR (Icobrain dm)
8. (JLK Inc.) OR JAD-02 K OR Atroscan
9. (Jung diagnostics) OR biometrica
10. mediaire OR mdbrain
11. Pixyl OR Neuro.BV
12. Quantib OR (Quantib ND)
13. Quibim OR (Quibim Precision)
14. Qynapse OR QYscore
15. (Siemens Healthineers) OR (AI-Rad Companion)
16. SyntheticMR OR (syMRI neuro)
17. Vuno OR (Vuno Med)

In conjunction, further relevant papers were searched through PubMed’s “related articles” function and cross-checking references from the initially identified studies and company websites. Finally, in order to capture studies published pre-branding, all vendors were contacted to provide further technical and clinical validation publications covering their QReports.

### Study inclusion criteria

Following steps 2–6 of the QNI six-step framework, the search terms described above were used to find peer-reviewed research covering technical and clinical validation, workflow integration and in-use evaluation for each QReport. Papers were reviewed for relevance and inclusion in our analysis on the basis that (i) they involve automated brain segmentation and volumetry results (ii) were published as original research in peer-reviewed academic journals or conference proceedings (conference posters were excluded) and (iii) fit into one of these four categories:

#### Technical validation

Papers presenting validation of the technical performance of brain segmentation technique and subsequent volumetric results, for example test–retest studies, standalone receiver operating characteristics or those comparing results (spatially and/or volumetrically) to manual segmentation and/or other state-of-the-art segmentation software, such as Freesurfer [32] or FSL-FIRST [33], regardless of disease area.

#### Clinical validation (dementia)

Testing the use of a QReport (tool meeting our inclusion criteria in “Vendor and product search” section) by clinicians (including but not limited to radiologists, neurologists, psychiatrists, neuropsychologists) on a dementia/memory clinic population within one or more of the following settings: (i) aiming to assess the QReport’s effect and impact on clinical management (i.e. usability and prognostic value); (ii) determining diagnostic accuracy, confidence, differential diagnoses vs. “ground truth” clinician-rated diagnoses, i.e. using receiver operating characteristics; (iii) percentage agreement or inter-rater reliability metrics; (iv) determining the correlation between automated volumetry and clinician-rated visual rating scales (e.g. MTA/Scheltens scale) and (v) clinical drug trials using the QReport’s results as an outcome measure in dementia trials.

#### Clinical validation (other neurological disease)

As above, but testing the use of a quantitative diagnostic report by clinicians in neurological diseases other than dementia or clinical drug trials using the QReport’s results as an outcome measure in trials of other neurological diseases.

While the focus of this review is dementia, it is also relevant to document the other instances where volumetric analysis methods from the vendors identified have been tested by clinician end-users, as this is ultimately the most critical phase of validation. Therefore, a few such examples found in the literature have been included in our analyses. It is of also interest to see how the various QReports have been used for research purposes alongside technical and clinical validation. However, these have not been included in the final results of our literature search because the focus of this review is validation, which should be most relevant to their clinical use, rather than examining the current range of their applicability in research.

#### Workflow integration and in-use evaluation

Papers analysing any of (i) benefit to patients; (ii) the effect on radiologist reporting time; (iii) clinical and population perception or (iv) the overall socioeconomic effect of using QReports in the clinic.

### Data extraction

All full-text articles evaluated that met the inclusion criteria were split into “Technical Validation”, “Clinical Validation—Dementia”, “Clinical Validation—Other” and “Workflow integration and in-use evaluation”, and were blindly assessed by two raters. The search and categorizing were replicated and verified by an independent researcher and no critical issues were detected. All relevant studies were categorized along with general information such as title, authors, year of publication, journal, associated tool and website. The technical information and features of the tools were also data based and are documented in Table 1.

**Table 1:**
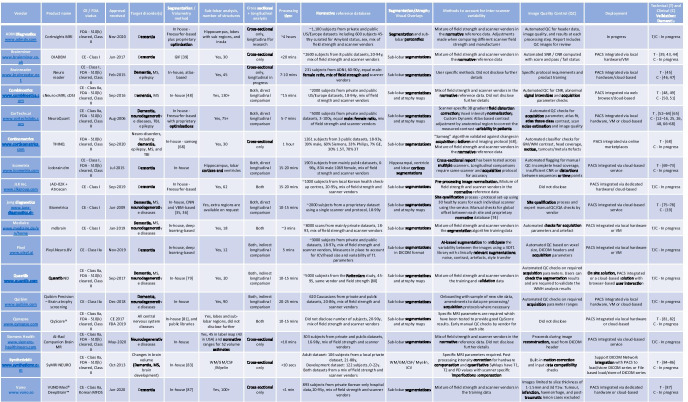
A high-level database of the vendors and various features in each of their QReports, presented in alphabetical order of vendor name. We have outlined information from publications and direct contact with vendors for readers to assess according to their individual needs. All information was checked and confirmed with vendors in advance of publication. Differing amounts of information between vendors is due to variation in how much the vendors were willing/able to share. Due to the proprietary nature of reports, it was not possible to independently verify all details from vendors but they were confirmed against sample reports where possible

Abbreviations: CNN, convolutional neural network; *VBM*, voxel-based morphometry; *SPM*, statistical parametric mapping; *GIF*, geodesic information flow; *TBI*, traumatic brain injury; *VM*, virtual machine; *GE*, general electric; *WMH*, white matter hyperintensity; *SNR*, signal to noise ratio; *CNR*, contrast to noise ratio; *QC*, quality control; *ICV*, intracranial volume; *PACS*, picture archiving and communication system

## Results

### Company and product search

Following the methods described above, 17 companies were identified that met our inclusion criteria. Each company had one QReport that met our inclusion criteria, see Fig. 1 for a research flow diagram summarizing the search for relevant products.

### Excluded tools

According to PRISMA guidelines, exclusion criteria were decided in advance of the systematic search and are listed in the “Methods” section. The various brain-related software tools that were excluded at the eligibility screening phase have been summarized below.

Tools not currently certified for clinical use were Imagilys (https://www.imagilys.com/), which is a previously CE-marked tool but their license recently expired. VEObrain produces a visual neuroradiological volumetry report but they have not yet been FDA/CE approved (https://www.veobrain.com/). Veganbagel (https://github.com/BrainImAccs/veganbagel) and volBrain (https://www.volbrain.upv.es/) are open-source software for estimation of regional brain volume changes and have been tested alongside visual rating scales [18, 21, 81]; veganbagel also has a PACS and workflow-integrated user interface. Freesurfer [32], FSL [33], VBM [66, 67] and SIENAX [82] are all well established and widely used brain research software but without clinical certification.

Tools requiring non-MRI input were eVox uses EEG to provide a map of brain function (https://evoxbrainmap.com/evox-brain-map/), Syntermed (https://www.syntermed.com/neuroq) and DOSISOFT (https://www.dosisoft.com/products/planet-neuro/) use FDG-PET to provide amyloid deposition maps.

Tools producing either non-volumetric reports or those focused on other neurological diseases were Advantis (https://advantis.io/) which offers 2D/3D visualization and post-processing workflows of DTI/tractography, DSC perfusion and fMRI.

Tools lacking normative reference data included QMENTA (https://www.qmenta.com/), a cloud-based application which accepts a broad range of MRI modalities and performs various statistical analyses. However, it provides no structured report or procedure for single-subject comparison to a normative reference population.

### Included tools

The companies and QReports identified through the search strategy detailed in the Methods section and illustrated in Fig. 1 are summarized in Table 1 along with technical details and features.

### Company and product features

Relevant information was compiled into Table 1, a structured database of the various information and features in each report. To complement Table 1, a general summary and some insight into the range of features recorded are outlined below.

#### CE/FDA approval status

All companies included in this review have received either CE class I/II marking or FDA 510 (k) clearance, as “software as a medical device”.

#### Date of approval

The first company (CorTechs.ai) received FDA clearance in 2006 and the most recent was certified in December 2020 (ADM diagnostics). Unsurprisingly, the older companies have generally published more peer-reviewed validation studies. It should be noted that all vendors have carried out internal technical validation processes, including the necessary steps for CE and/or FDA clearance. All companies contacted, and especially the younger ones, claimed to be planning further peer-reviewed validation studies.

#### Report processing time

A wide array of QReport processing times were reported across the vendors ranging from a few seconds to a few hours, which is highly dependent on local vs cloud-based deployment. It should be noted that we were unable to verify the reported times without access to each of the software packages.

#### Segmentation/volumetry method

The vast majority of companies use proprietary methods developed “in house”, of which five claim to use deep learning. Several companies have used modified versions of previously reported research methods, such as geodesic information flows (GIF) [34, 83], Freesurfer [32] and VBM [66].

#### Sub-regional volumetry

All vendors provide lobar and hippocampal volumetry as a minimum. Beyond these regions, companies range from adding only ventricular information to providing over 100 sub-lobar regions as part of their structured reports. Some companies reported excluding various sub-lobar regions due to reproducibility issues and others claimed extensive reporting of such regions was not of interest to their users.

#### Cross-sectional and longitudinal analyses

Ten companies provide both cross-sectional and longitudinal analyses. Longitudinal comparisons were broadly indirect approaches, i.e. the difference in volume/percentile per structure between two visits, rather than a direct approach such as the boundary shift integral [84–86] or SIENA [82].

#### Details of a normative reference population

Some of the most notable variations across companies is seen in the number, age range and breadth of subjects/data used in the normative reference population. The vast majority of vendors reported a mix of gender, scanner type and field strength achieved through the use of both private and public datasets. However, the size of the dataset varied greatly from ~ 100 to ~ 8000. The age ranges were more consistent and broadly covered the 20–90 years range.

#### Target disorder

All companies reported dementia as a target disorder. Eleven tools were said to be aimed at multiple disorders, including epilepsy, traumatic brain injury and MS, in addition to dementia.

#### Provision of cortical overlays/atrophy heat maps

All companies provide some form of cortical overlay back to the user. These were either segmentation examples for accuracy confirmation, atrophy-based heat maps or both.

#### Image quality control (QC) method

Techniques for image QC before report processing varied greatly, ranging from specific acquisition protocol requirements to automated artefact checks and automated flagging for manual QC.

#### Strategies to account for inter-scanner variability

All companies informed us that harmonization measures were in place, although some declined to provide proprietary details. The type of strategy varies considerably, including an equal mix of field strength, scanner vendor and acquisition parameters in the reference dataset; vendor-specific acquisition parameters and site qualification procedures; and adopting validated variation-agnostic segmentation algorithms.

#### PACS integration/report deployment procedure

All companies claimed to provide PACS integration of their tools, some offer web-based, cloud-based or separate hardware solutions.

#### Peer-reviewed technical and clinical validation

**Fig. 2:**
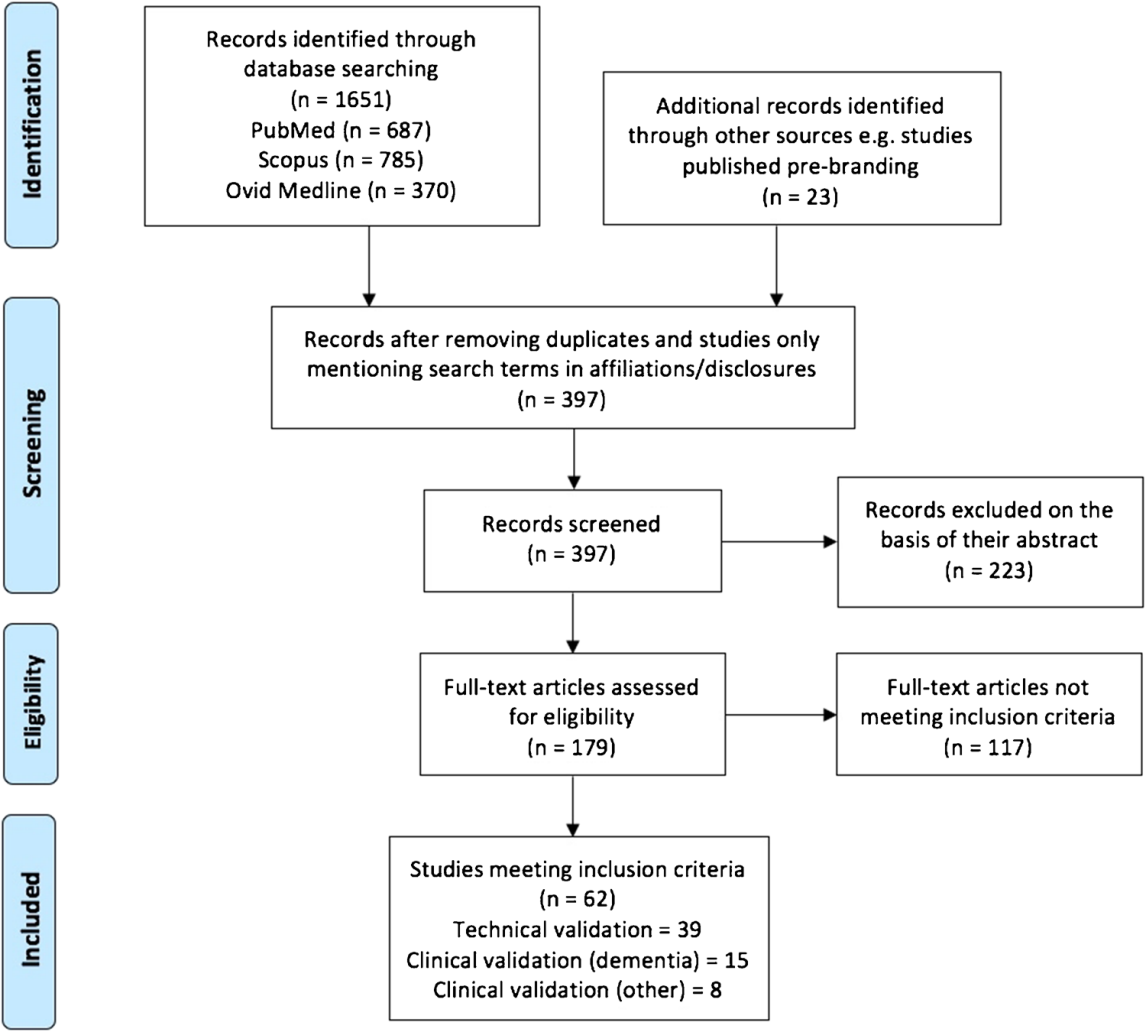
PRISMA flowchart documenting the studies searched and selected for inclusion in this review

The number and category of studies found during this systematic literature review are presented in Fig. 2 and the “Literature Search” section.

### Literature search

The literature search, screening, final selection and categorization were conducted in line with the PRISMA guidelines [28–30]; the results are outlined in a PRISMA workflow diagram (Fig. 2) and documented further below. A total of 62 original studies covering technical (39) or clinical validation (23, dementia = 15, other neurological diseases = 8) were identified from 11 of the 17 companies/products assessed. For 6 products, no publications meeting our inclusion criteria were identified. Only 4 vendors have published clinical validation of their reports in a dementia population.

**Fig. 3:**
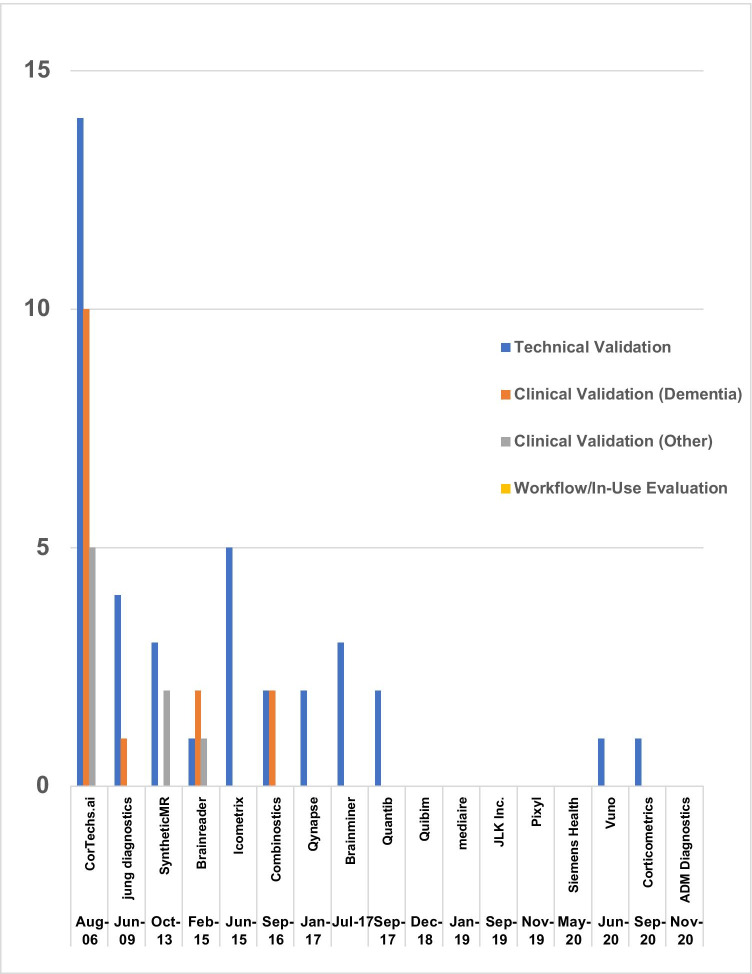
The distribution of papers meeting our inclusion criteria for each of the companies identified. The vendors are listed in chronological order according to the date of their first CE/FDA approval

The distribution of studies identified is shown in Fig. 3. As expected, there was considerable variation amongst the vendors in the number and type of validation studies performed. However, all companies claimed to be planning further peer-reviewed validation studies.

### Validation studies identified

Of the 17 companies assessed, 11 have published some form of technical validation on their segmentation methods; only 4 have published clinical validation of their QReport in a dementia population and 3 when using the same report in other neurodegenerative disorders, totalling 62 studies. It should be noted that all QReports identified have satisfied the validation requirements for FDA clearance and/or CE marking. However, these markings do not guarantee diagnostic value; further rigorous independent validation studies should be conducted and published in peer-reviewed journals to assist potential users’ decision-making between available tools. In order to remain unbiased, a narrative synthesis of the various studies searched for each company is provided and referenced below (in alphabetical order). In general, more technical than clinical validation has been published by companies and research groups using proprietary QReports. Technical validation studies broadly reported strong correlation between automated segmentations and that of manual raters or state-of-the-art research tools, such as Freesurfer. Clinical validation studies of quantitative reports on dementia patients, albeit scarce, conveyed improved diagnostic accuracy [38, 58], prognostic value [39, 57], differential diagnosis [19] and confidence [42] amongst clinicians or vs. clinician diagnoses, as well as strong correlation with the diagnostic potential of visual rating scales [43, 59, 87].

#### Brainminer:

DIADEM uses the geodesic information flows (GIF) methodology for brain segmentation and volumetry, which has been tested [34] against the MAPER segmentation technique [88]. GIF has also previously been tested against manual segmentations [35, 36].

#### Brainreader:

Volumetry results from the Neuroreader report have been compared to manual segmentations [37]. **Clinical:** Automated hippocampal volumes were compared to NeuroQuant’s in terms of predicting conversion from mild cognitive impairment (MCI) to AD [39]. Radiologists have tested the validity of Neuroreader for detecting mesial temporal sclerosis in epilepsy patients [89] and dementia diagnosis in a memory clinic cohort [38].

#### Combinostics:

Combinostics’ segmentation method has been compared to manual segmentations [40] and tested for standalone disease classification [90]. **Clinical:** The performance of their automatically generated MTA and GCA rating scales has been compared to radiologists’ assessment [43]. The PredictND tool for prognostic assessment has been tested by a clinician [42].

#### CorTechs.ai:

Automated segmentations have been both manually checked and compared to manual segmentations [44, 45, 47, 52, 55], FreeSurfer [46, 50–52, 56, 57], FSL-FIRST [47, 53], SIENAX [48] and other FDA/CE-marked tools: MSmetrix [48]. One study also assessed the difference in results following a version update [49]. Furthermore, a new MR volumetry software (Inbrain—https://www.inbrain.co.kr/) recently compared their results to NeuroQuant [54]. **Clinical:** NeuroQuant has been used by radiologists in the context of traumatic brain injury [25, 91], temporal lobe epilepsy [92–94] and AD [58, 59, 87]. The prognostic value of NeuroQuant has been assessed in MCI patients [39, 57]. NeuroQuant’s volumetry results have been used as an outcome measure in a number of dementia-related clinical trials, covering immunoglobulin [12], Ab immunotherapy CAD106 [13], resveratrol [14], 8-OH quinoline [15] and adipose-derived stromal vascular fraction [16].

#### Corticometrics:

The THINQ report uses the segmentation and volumetry method samseg, which has been tested in one study [60] alongside multi-atlas likelihood fusion (PICSL-MALF) [95], Brainfuse [96], majority voting [97] and Freesurfer.

#### Icometrix:

Volumetric results from icobrain dm were recently compared to Freesurfer [62]. The longitudinal comparison tool, icobrain long, has also been tested against SIENAX with real-world MS data [98]. Their MS-specific report, MSmetriX, which uses the same volumetry technique, has been tested intercontinentally [65] and validated against SIENA on MS [63] and AD patients [64].

#### jung diagnostics:

The Biometrica platform uses the widely validated SPM for volumetry [70] and has been compared to the SIENA and FSL tools [71, 72]. Hippocampal segmentations have previously been verified by radiologists [69]. **Clinical:** The Biometrica report’s effect on dementia diagnosis has also been tested by neuroradiologists [19].

#### Quantib:

Quantib’s segmentation method has previously been compared with manual segmentations [73, 99].

#### Qynapse:

The Qynapse segmentation method has been tested against manual segmentations [75, 76].

#### SyntheticMR:

SyMRI’s volumetry results have been assessed in a repeatability studies and manual segmentation study [100]. The automated brain parenchymal fraction generator has been compared with manual techniques, VBM8 and SPM12, in MS patients [79] and healthy controls [78]. **Clinical:** The SyMRI report results were used in a clinical trial of rituximab on MS patients [17].

#### Vuno:

Vuno’s deep learning segmentation methods have been tested for standalone disease classification [80].

## Discussion

In this systematic review, we have identified a broad range of companies offering CE-marked or FDA-cleared QReports for use in dementia populations. The available publications concerning technical and clinical validation of these tools were categorized to increase the transparency of evidence. However, product ranking or recommendations have been avoided due to variations in the needs of each purchaser and user. Beyond regulatory body approval, QReports on the market vary widely in how they have been technically and clinically validated for use in clinical practice. Of the 17 companies assessed, 11 have published some form of technical validation on their segmentation methods; only 4 have published clinical validation of their QReports in a dementia population and 3 when using the same report in other neurodegenerative disorders. For 6 products, no publications were found that met our inclusion criteria. We found no published evidence for any regulatory approved QReports on workflow integration or in-use evaluation, as recommended in steps 5 and 6 of the QNI framework. However, all vendors informed us that they are planning (further) validation studies. It is worth noting that the European Medical Devices Regulation has recently implemented a “post-market clinical follow-up” in conjunction with their “post-market surveillance” and “clinical evaluation reporting” (https://ec.europa.eu/health/md_sector/overview_en). This will require vendors to gather, record and analyse their clinical performance and safety data throughout the lifecycle of their product in order to achieve certification or re-certification. Hopefully, this will stimulate the publication of external peer-reviewed validation studies by vendors.

Previously published reviews covering quantitative radiological tools have either focused purely on AI-driven image analysis software for broader radiology [101–103] or only covered a limited number of tools available on the market focused on neuropsychiatry [104, 105]. In recent years, there has been a considerable rise in companies providing both AI and non-AI-based automated quantitative analysis methods: 12 of the 17 identified in this study are less than 3 years old. This growth recently prompted the FDA to produce an “action plan” for AI/machine learning-based software as a medical device—https://www.fda.gov/media/145022/download. In this paper, they outline plans to update current regulatory frameworks, strengthen the harmonized development of “good machine learning practice”, support a patient-centred approach and, most relevant to this review, support the development of methods for evaluating and improving machine learning algorithms and promote real-world performance studies, in other words, technical and clinical validation. The ECLAIR guidelines were also published very recently aiming to provide guidance and informed decision-making when evaluating commercial AI solutions in radiology before purchase [106].

Using structured and validated QReports could provide considerable improvements in diagnostic accuracy, reliability, confidence and efficiency across a neuroradiological service but is predicated upon technical and clinical validation [2, 8, 21, 107, 108]. Previous research has shown that these diagnostic improvements could be achieved by providing region-specific volumetric differences between single-subjects and an age-matched normative population [18–23, 91, 109–111]. Work to this effect has been underway for some time but there is currently no rigorously validated platform for automated quantification and display of volumetric data in widespread use for radiology reporting. There are several hurdles for clinical implementation of volumetric analysis, such as a discrepancy in the quality of research and clinical data, need for automated detection of image artefacts, inter-scanner variability and the requirement of full automation. Indeed, only 23% of 193 centres assessed in a recent European survey performed volumetric analysis, and only 5.7% reported using it regularly [27]. Of the 23% using volumetry, only around half used normative reference data for single-subject comparison. The majority of centres reported using FreeSurfer (43.5%) for volumetric processing, followed by CorTechs.ai’s NeuroQuant (17.4%), AppMRI hippocampus volume analyser (15.2%) and Icometrix (4.3%). It is notable that the highest percentage of reported use of a clinical proprietary tool (17.4%) was exhibited by NeuroQuant, which is also the tool that has been most widely validated thus far. It follows that extensive technical and clinical validation of the tools described in this review will likely increase user confidence and facilitate the adoption of quantitative methods in the clinic.

The features offered by the QReports identified vary widely, see Table 1. No “one-size-fits-all” approach exists for the complex requirements of each clinician, department or patient population. The same applies to the degree and type of validation in the peer-reviewed literature: studies relevant to one population may be less so to another. In order to remain unbiased, a summary of QReport features and validation studies in the literature has been provided but detailed study results and product recommendations are avoided due to the variation in the needs of each purchaser and user. Indeed, the selection of QReports depends on several factors, such as resources, experience and expertise already available in a clinical group, product regulation, technical and clinical validation, generalisability to the patient population seen in clinic, integration of software into the clinical workflow, customer support, data security requirements and cost/return on investment/reimbursement eligibility. It was not possible to gather purchase costs for this review but a recent overview of volumetric quantification in neurocognitive disorders reported costs on average to be USD82.68 per patient [105]. However, the actual costs of implementing these tools in a clinic may vary by a country where the healthcare system, reimbursement regulations and healthcare costs all playing a role.

### What evidence would an ideal QReport exhibit on the way to clinical integration?

A six-step framework for the translation of clinical reporting tools has been previously set out by the QNI [2]. Here we discuss some of the most important milestones in the development of a dementia-specific QReport. The main aspect and the focus of this review is the transparency of technical and clinical validation as this should be of the utmost importance to end-users and critical to ensuring patient benefit.

#### Technical validation vs industry standards

Any QReports intended for use as a diagnostic aid in neurodegenerative diseases should communicate both patient and normative volumetric results via a visually intuitive and clinically relevant report. Ideally, we suggest that this should include automated quality control metrics, cortical overlays of the segmentation for sanity checking by the end-user and visual representation of the quantitative data in a graph or chart and/or atrophy-based heat maps for easy reference. The automated segmentation method should undergo rigorous technical validation in repeatability studies and versus industry standards such as expert manual segmentation, Freesurfer, FSL or VBM, and the results published in peer-reviewed journals. All the vendors assessed in this review have produced quantitative reports to assist volumetric MRI analysis. However, the younger companies are generally have not published technical validation of their reports, although all claimed to be planning.

#### Clinical validation by end-users

Several papers assess the predictive capability of tools for automated group-level differential diagnoses amongst dementia subtypes in a research setting [59, 80, 90, 112–115]. However, the purpose of this review is to help clinicians select the most appropriate tools for their individual investigations in everyday clinical practice. Automated group-level diagnosis studies without intervention and testing by end-users are far less relevant to the clinic. QReports should be tested by the end-users, usually clinicians, on multi-centre clinical data from patient populations that are expected to benefit most from more accurate and faster diagnoses. For example, screening for subjective memory concerns and diagnoses for younger onset dementia patients. These patient populations may have more subtle patterns of atrophy and QReports are likely to provide the greatest benefit to raters by flagging patients who require more regular follow-ups and reducing inter-rater variability. The results of diagnostic accuracy studies are ideally published in peer-reviewed journals [19–21, 116–118]. Several companies provide lists of publications on their website. While this is both positive and helpful, direct references to technical and clinical validation of QReports are scarce. For the greatest impact and widest adoption of these tools, peer-reviewed validation studies should be clearly highlighted and championed by vendors. While technical validation has been covered by 11 of 17 vendors, only 4 have published clinical validation of their tools on a dementia or memory clinic population. We have identified a major lack of clinical validation studies for volumetric neuroradiological tools in the literature.

#### Proven generalisability

Analysis methods should ideally be robust to variation in acquisition parameters, scanner/vendor differences and field strength, although this is a difficult standard to achieve in reality. Single-subject results should be contextualized against a large and generalizable reference population of mixed field strengths, scanner vendors and age and gender-matched controls, ideally transferrable to the demographic of patients that will be seen in each clinic. For example, a tool using a reference population comprised of data purely from an Asian hospital might not translate well for use at a clinic based in Europe or the Americas. Limited evidence so far suggests that mean subcortical volumes in normative cohorts have proven to be reasonably interchangeable across reference populations [111], though this needs further support from studies with multi-ethnic populations and covering more brain regions. In general, vendors have compiled sufficiently large and diverse normative reference populations and should continue to be transparent about the source and composition of these cohorts. However, as documented in the Results section, there is wide variation in generalisability procedures adopted by companies. There is no single universally accepted or correct method but companies should be fully transparent regarding the measures they have in place to account for the variability of input data.

#### Full automation and workflow integration

This covers step 5 in the QNI framework. Vendors should be able to provide clear methods for PACS and workflow integration and ideally full automation of sending scans for processing and receiving results. Furthermore, a system for integrating QReport results into the radiologist’s report would save time and reduce copying errors. Customer support operations must also be in place to deal with errors in sending and processing. While many tools reviewed here do include methods to accommodate workflow integration, we found no research evidence regarding the integration of QReports into the clinical reporting workflow.

#### In-use evaluation

This covers step 6 in the QNI framework but, like step 5, the literature review did not uncover any evidence of in-use evaluation of the QReports included in this paper. However, work has been presented to map out the relevance of automated software for radiology in general [119–121]. While the benefit to patients should be the key factor in using automated volumetry to assist diagnosis, the socioeconomic impact, while heavily associated with patient benefit, should also be assessed. Multi-centre studies evaluating clinical and population perception and cost-effectiveness of quantitative report use should be conducted in clinics that have been regularly using reports for a sufficient period of time.

## Limitations

Some limitations of the current review need to be considered. In order to find as many companies providing QReports, an extensive FDA/CE approval search was conducted. However, without a fully searchable database of CE-marked products, this approach may not be fully exhaustive and some vendors could have been missed. Furthermore, some products may have received regulatory approval during the publication process of this manuscript or have been approved for other markets. Despite that, our overall conclusion remains unchanged that there is a need for more clinical validation for such tools to facilitate optimal clinical adoption. Especially since we found that the younger vendors were most lacking in both technical and clinical validation and in-use evaluation. Finally, much of the information on the features of each company (see Table 1) was provided by the vendors themselves. As such, these details could not all be independently verified by the authors or the reviewers.

### Future developments

While we have focused primarily on evidence of technical and clinical validation of QReports, we also observed wide variation in capabilities across tools and in the information presented. Conducting in-use evaluations, as recommended in step six of the QNI framework, will help optimize the functions, features and design of QReports based on how they foster clinical efficacy. Another natural progression from this conclusion would be to present a side-by-side comparison of each of the reports and their results including interpretation by radiologists and their clinical impact using a test set of subjects from the same dataset, such as ADNI or a real-world dataset reflecting everyday clinical practice. Eleven of the 17 companies covered in this study told us that they would be willing to participate in such a project.

## Conclusions

In this review, we reveal a significant evidence gap in the clinical validation of QReports for use in dementia diagnosis and memory clinic settings. Only 4 of the 17 companies assessed have so far published some kind of clinical validation and there is not yet any evidence of workflow integration nor in-use evaluation. From this, we conclude and recommend that more research can be done to validate these QReports in clinical settings to develop a more robust understanding of how each tool contributes to the diagnostic workflow in memory clinics. This will not only support optimal clinical integration of quantitative tools but will also help neuroradiologists to make informed decisions regarding the use of quantitative assessment in their clinics. For clinicians interested in incorporating quantitative reporting software into their diagnostic workflow, note that while 4 companies have published clinical validation studies, owing to large variation in the quantitative reporting features available and a lack of comparative validation on standardized imaging cohort data, there is little scope for recommendation between them with regard to their utility as diagnostic tools in the clinic. We hope this review encourages such validation studies from the developers of these quantitative tools and recommend caution from clinicians when examining claims of the tools’ clinical performance.

## Data Availability

This review paper covers public data and data provided by the companies.

## Abbreviations

AD: Alzheimer’s disease
CE: *Conformité Européenne*
FDA: Food and Drug Administration
GCA: Global cortical atrophy
MRI: Magnetic resonance imaging
MTA: Medial temporal atrophy
PRISMA: Preferred Reporting Items for Systematic Reviews and Meta-Analyses
PROSPERO: Prospective Register of Systematic Reviews
QC: Quality control
QNI: Quantitative neuroradiology initiative
QReport: Quantitative volumetric report

## Code availability

The script for keyword searching in the FDA database is available—https://github.com/hughp1/FDA_KeyWordSearch/tree/main.

## References

[1] Risacher SL, Saykin AJ (2013). Neuroimaging biomarkers of neurodegenerative diseases and dementia. Semin Neurol.

[2] Goodkin O, Pemberton H, Vos SB (2019). The quantitative neuroradiology initiative framework: application to dementia. Br J Radiol.

[3] Vernooij MW, Smits M (2012). Structural neuroimaging in aging and Alzheimer’s disease. Neuroimaging Clin N Am.

[4] Scheltens P, Leys D, Barkhof F (1992). Atrophy of medial temporal lobes on MRI in &quot;probable&quot; Alzheimer’s disease and normal ageing: diagnostic value and neuropsychological correlates. J Neurol Neurosurg Psychiatry.

[5] Boutet C, Chupin M, Colliot O (2012). Is radiological evaluation as good as computer-based volumetry to assess hippocampal atrophy in Alzheimer’s disease?. Neuroradiology.

[6] Pereira JB, Cavallin L, Spulber G (2014). Influence of age, disease onset and *ApoE4* on visual medial temporal lobe atrophy cut-offs. J Intern Med.

[7] ten Kate M, Barkhof F, Boccardi M, et al (2017) Clinical validity of medial temporal atrophy as a biomarker for Alzheimer’s disease in the context of a structured 5-phase development framework. Neurobiol Aging 10.1016/j.neurobiolaging.2016.05.024 28317647

[8] McEvoy LK, Brewer JB (2010). Quantitative structural MRI for early detection of Alzheimer’s disease. Expert Rev Neurother.

[9] Frost C, Kenward MG, Fox NC (2004). The analysis of repeated ‘direct’ measures of change illustrated with an application in longitudinal imaging. Stat Med.

[10] Schwarz AJ, Sundell KL, Charil A (2019). Magnetic resonance imaging measures of brain atrophy from the EXPEDITION3 trial in mild Alzheimer’s disease. Alzheimer’s Dement Transl Res Clin Interv.

[11] Salvatore C, Cerasa A, Castiglioni I (2018). MRI characterizes the progressive course of AD and predicts conversion to Alzheimer’s dementia 24 months before probable diagnosis. Front Aging Neurosci.

[12] Relkin NR, Thomas RG, Rissman RA (2017). A phase 3 trial of IV immunoglobulin for Alzheimer disease. Neurology.

[13] Vandenberghe R, Riviere ME, Caputo A (2017). Active Aβ immunotherapy CAD106 in Alzheimer’s disease: a phase 2b study. Alzheimer’s Dement Transl Res Clin Interv.

[14] Turner RS, Thomas RG, Craft S (2015). A randomized, double-blind, placebo-controlled trial of resveratrol for Alzheimer disease. Neurology.

[15] Villemagne VL, Rowe CC, Barnham KJ (2017). A randomized, exploratory molecular imaging study targeting amyloid β with a novel 8-OH quinoline in Alzheimer’s disease: the PBT2-204 IMAGINE study. Alzheimer’s Dement Transl Res Clin Interv.

[16] Duma C, Kopyov O, Kopyov A (2019). Human intracerebroventricular (ICV) injection of autologous, non-engineered, adipose-derived stromal vascular fraction (ADSVF) for neurodegenerative disorders: results of a 3-year phase 1 study of 113 injections in 31 patients. Mol Biol Rep.

[17] Salzer J, Svenningsson R, Alping P (2016). Rituximab in multiple sclerosis. Neurology.

[18] Hedderich DM, Spiro JE, Goldhardt O (2018). Increasing diagnostic accuracy of mild cognitive impairment due to Alzheimer’s disease by user-independent, web-based whole-brain volumetry. J Alzheimer’s Dis.

[19] Hedderich DM, Dieckmeyer M, Andrisan T (2020). Normative brain volume reports may improve differential diagnosis of dementing neurodegenerative diseases in clinical practice. Eur Radiol.

[20] Pemberton HG, Goodkin O, Prados F, et al (2021) Automated quantitative MRI volumetry reports support diagnostic interpretation in dementia: a multi-rater, clinical accuracy study. Eur Radiol 1–12. 10.1007/s00330-020-07455-8 10.1007/s00330-020-07455-8 PMC8213665 33452627

[21] Caspers J, Heeger A, Turowski B, Rubbert C (2020). Automated age- and sex-specific volumetric estimation of regional brain atrophy: workflow and feasibility. Eur Radiol.

[22] Vernooij MW, Jasperse B, Steketee R (2018). Automatic normative quantification of brain tissue volume to support the diagnosis of dementia: a clinical evaluation of diagnostic accuracy. NeuroImage Clin.

[23] Klöppel S, Yang S, Kellner E (2018). Voxel-wise deviations from healthy aging for the detection of region-specific atrophy. NeuroImage Clin.

[24] Brewer JB, Magda S, Airriess C, Smith ME (2009). Fully-automated quantification of regional brain volumes for improved detection of focal atrophy in Alzheimer disease. Am J Neuroradiol.

[25] Ross DE, Ochs AL, DeSmit ME (2015). Man versus machine part 2: comparison of radiologists’ interpretations and NeuroQuant measures of brain asymmetry and progressive atrophy in patients with traumatic brain injury. J Neuropsychiatry Clin Neurosci.

[26] Ross DE, Ochs AL, Seabaugh JM, Shrader CR (2013). Man versus machine: comparison of radiologists’ interpretations and NeuroQuantspi® volumetric analyses of brain MRIs in patients with traumatic brain injury. J Neuropsychiatry Clin Neurosci.

[27] Vernooij MW, Pizzini FB, Schmidt R (2019). Dementia imaging in clinical practice: a European-wide survey of 193 centres and conclusions by the ESNR working group. Neuroradiology.

[28] Liberati A, Altman DG, Tetzlaff J, et al (2009) The PRISMA statement for reporting systematic reviews and meta-analyses of studies that evaluate healthcare interventions: explanation and elaboration. BMJ 339. 10.1136/bmj.b2700 10.1136/bmj.b2700 PMC2714672 19622552

[29] Moher D, Liberati A, Tetzlaff J, Altman DG (2009). Preferred reporting items for systematic reviews and meta-analyses: the PRISMA statement. BMJ.

[30] Moher D, Shamseer L, Clarke M (2016). Preferred reporting items for systematic review and meta-analysis protocols (PRISMA-P) 2015 statement. Rev Esp Nutr Humana y Diet.

[31] Overview | Public Health. https://ec.europa.eu/health/md_eudamed/overview_en. Accessed 28 Mar 2021

[32] Fischl B (2012). FreeSurfer. Neuroimage.

[33] Smith SM, Jenkinson M, Woolrich MW, et al (2004) Advances in functional and structural MR image analysis and implementation as FSL. In: NeuroImage. Neuroimage 10.1016/j.neuroimage.2004.07.051 15501092

[34] Cardoso MJ, Modat M, Wolz R (2015). Geodesic information flows: spatially-variant graphs and their application to segmentation and fusion. IEEE Trans Med Imaging.

[35] de Sitter A, Verhoeven T, Burggraaff J (2020). Reduced accuracy of MRI deep grey matter segmentation in multiple sclerosis: an evaluation of four automated methods against manual reference segmentations in a multi-center cohort. J Neurol.

[36] Bocchetta M, Iglesias JE, Russell LL, et al (2019) Segmentation of medial temporal subregions reveals early right-sided involvement in semantic variant PPA. Alzheimer’s Res Ther 11. 10.1186/s13195-019-0489-9 10.1186/s13195-019-0489-9 PMC6511178 31077248

[37] Ahdidan J, Raji CA, DeYoe EA (2015). Quantitative neuroimaging software for clinical assessment of hippocampal volumes on MR imaging. J Alzheimer’s Dis.

[38] Morin A, Samper-Gonzalez J, Bertrand A (2020). Accuracy of MRI classification algorithms in a Tertiary Memory Center Clinical Routine Cohort. J Alzheimer’s Dis.

[39] Tanpitukpongse TP, Mazurowski MA, Ikhena J, Petrella JR (2017). Predictive utility of marketed volumetric software tools in subjects at risk for Alzheimer disease: do regions outside the hippocampus matter?. Am J Neuroradiol.

[40] Lötjönen JM, Wolz R, Koikkalainen JR (2010). Fast and robust multi-atlas segmentation of brain magnetic resonance images. Neuroimage.

[41] Tolonen A, Rhodius-Meester HFM, Bruun M, et al (2018) Data-driven differential diagnosis of dementia using multiclass disease state index classifier. Front Aging Neurosci 10. 10.3389/fnagi.2018.00111 10.3389/fnagi.2018.00111 PMC5996907 29922145

[42] Bruun M, Frederiksen KS, Rhodius-Meester HFM (2019). Impact of a clinical decision support tool on prediction of progression in early-stage dementia: a prospective validation study. Alzheimers Res Ther.

[43] Koikkalainen JR, Rhodius-Meester HFM, Frederiksen KS (2019). Automatically computed rating scales from MRI for patients with cognitive disorders. Eur Radiol.

[44] Brewer JB, Magda S, Airriess C, Smith ME (2009) Fully-automated quantification of regional brain volumes for improved detection of focal atrophy in Alzheimer disease. Am J Neuroradiol. 10.3174/ajnr.A1402 10.3174/ajnr.A1402 PMC5947999 19112065

[45] Kovacevic S, Rafii MS, Brewer JB (2009). High-throughput, fully automated volumetry for prediction of MMSE and CDR decline in mild cognitive impairment. Alzheimer Dis Assoc Disord.

[46] Ochs AL, Ross DE, Zannoni MD (2015). Comparison of automated brain volume measures obtained with NeuroQuant® and FreeSurfer. J Neuroimaging.

[47] Lyden H, Gimbel SI, Del Piero L, et al (2016) Associations between family adversity and brain volume in adolescence: manual vs. automated brain segmentation yields different results. Front Neurosci 10. 10.3389/fnins.2016.00398 10.3389/fnins.2016.00398 PMC5011142 27656121

[48] Wang C, Beadnall HN, Hatton SN (2016). Automated brain volumetrics in multiple sclerosis: a step closer to clinical application. J Neurol Neurosurg Psychiatry.

[49] Stelmokas J, Yassay L, Giordani B (2017). Translational MRI volumetry with NeuroQuant: effects of version and normative data on relationships with memory performance in healthy older adults and patients with mild cognitive impairment. J Alzheimer’s Dis.

[50] Reid MW, Hannemann NP, York GE (2017). Comparing two processing pipelines to measure subcortical and cortical volumes in patients with and without mild traumatic brain injury. J Neuroimaging.

[51] Ross DE, Ochs AL, Tate DF (2018). High correlations between MRI brain volume measurements based on NeuroQuant® and FreeSurfer. Psychiatry Res - Neuroimaging.

[52] Brinkmann BH, Guragain H, Kenney-Jung D (2019). Segmentation errors and intertest reliability in automated and manually traced hippocampal volumes. Ann Clin Transl Neurol.

[53] Pareto D, Sastre-Garriga J, Alberich M (2019). Brain regional volume estimations with NeuroQuant and FIRST: a study in patients with a clinically isolated syndrome. Neuroradiology.

[54] Lee JY, Oh SW, Chung MS, et al (2020) Clinically available software for automatic brain volumetry: comparisons of volume measurements and validation of intermethod reliability. Korean J Radiol 21. 10.3348/kjr.2020.0518 10.3348/kjr.2020.0518 PMC7909859 33236539

[55] Feng CH, Cornell M, Moore KL, et al (2020) Automated contouring and planning pipeline for hippocampal-avoidant whole-brain radiotherapy. Radiat Oncol 15. 10.1186/s13014-020-01689-y 10.1186/s13014-020-01689-y PMC7602303 33126894

[56] Yim Y, Lee JY, Oh SW (2021). Comparison of automated brain volume measures by NeuroQuant vs. Freesurfer in patients with mild cognitive impairment: effect of slice thickness. Yonsei Med J.

[57] Yu P, Sun J, Wolz R (2014). Operationalizing hippocampal volume as an enrichment biomarker for amnestic mild cognitive impairment trials: effect of algorithm, test-retest variability, and cut point on trial cost, duration, and sample size. Neurobiol Aging.

[58] Persson K, Barca ML, Cavallin L (2018). Comparison of automated volumetry of the hippocampus using NeuroQuant® and visual assessment of the medial temporal lobe in Alzheimer’s disease. Acta radiol.

[59] Persson K, Selbæk G, Brækhus A (2017). Fully automated structural MRI of the brain in clinical dementia workup. Acta radiol.

[60] Puonti O, Iglesias JE, Van Leemput K (2016). Fast and sequence-adaptive whole-brain segmentation using parametric Bayesian modeling. Neuroimage.

[61] Beadnall HN, Wang C, Van Hecke W (2019). Comparing longitudinal brain atrophy measurement techniques in a real-world multiple sclerosis clinical practice cohort: towards clinical integration?. Ther Adv Neurol Disord.

[62] Struyfs H, Sima DM, Wittens M (2020). Automated MRI volumetry as a diagnostic tool for Alzheimer’s disease: validation of icobrain dm. NeuroImage Clin.

[63] Smeets D, Ribbens A, Sima DM, et al (2016) Reliable measurements of brain atrophy in individual patients with multiple sclerosis. Brain Behav 6. 10.1002/brb3.518 10.1002/brb3.518 PMC5036437 27688944

[64] Niemantsverdriet E, Ribbens A, Bastin C (2018). A retrospective Belgian Multi-Center MRI Biomarker Study in Alzheimer’s disease (REMEMBER). J Alzheimer’s Dis.

[65] Finkelsztejn A, Fragoso YD, Bastos EA (2018). Intercontinental validation of brain volume measurements using MSmetrix. Neuroradiol J.

[66] Good CD, Johnsrude IS, Ashburner J (2001). A voxel-based morphometric study of ageing in 465 normal adult human brains. Neuroimage.

[67] Frisoni GB, Testa C, Sabattoli F (2005). Structural correlates of early and late onset Alzheimer’s disease: voxel based morphometric study. J Neurol Neurosurg Psychiatry.

[68] Schippling S, Ostwaldt AC, Suppa P (2017). Global and regional annual brain volume loss rates in physiological aging. J Neurol.

[69] Suppa P, Hampel H, Spies L (2015). Fully automated atlas-based hippocampus volumetry for clinical routine: validation in subjects with mild cognitive impairment from the ADNI cohort. J Alzheimer’s Dis.

[70] Opfer R, Suppa P, Kepp T (2016). Atlas based brain volumetry: how to distinguish regional volume changes due to biological or physiological effects from inherent noise of the methodology. Magn Reson Imaging.

[71] Opfer R, Ostwaldt AC, Walker-Egger C (2018). Within-patient fluctuation of brain volume estimates from short-term repeated MRI measurements using SIENA/FSL. J Neurol.

[72] Opfer R, Ostwaldt AC, Sormani MP (2018). Estimates of age-dependent cutoffs for pathological brain volume loss using SIENA/FSL—a longitudinal brain volumetry study in healthy adults. Neurobiol Aging.

[73] de Boer R, Vrooman HA, Ikram MA (2010). Accuracy and reproducibility study of automatic MRI brain tissue segmentation methods. Neuroimage.

[74] Ikram MA, van der Lugt A, Niessen WJ (2015). The Rotterdam Scan Study: design update 2016 and main findings. Eur J Epidemiol.

[75] Chupin M, Mukuna-Bantumbakulu AR, Hasboun D, et al (2007) Anatomically constrained region deformation for the automated segmentation of the hippocampus and the amygdala: method and validation on controls and patients with Alzheimer’s disease. Neuroimage 34. 10.1016/J.NEUROIMAGE.2006.10.035 10.1016/j.neuroimage.2006.10.035 17178234

[76] Chupin M, Gérardin E, Cuingnet R, et al (2009) Fully automatic hippocampus segmentation and classification in Alzheimer’s disease and mild cognitive impairment applied on data from ADNI. Hippocampus 19. 10.1002/HIPO.20626 10.1002/hipo.20626 PMC2837195 19437497

[77] West J, Warntjes JBM, Lundberg P (2012). Novel whole brain segmentation and volume estimation using quantitative MRI. Eur Radiol.

[78] Vågberg M, Ambarki K, Lindqvist T (2016). Brain parenchymal fraction in an age-stratified healthy population – determined by MRI using manual segmentation and three automated segmentation methods. J Neuroradiol.

[79] Vågberg M, Lindqvist T, Ambarki K (2013). Automated determination of brain parenchymal fraction in multiple sclerosis. Am J Neuroradiol.

[80] Suh CH, Shim WH, Kim SJ, et al (2020) Development and validation of a deep learning–based automatic brain segmentation and classification algorithm for Alzheimer disease using 3D T1-weighted volumetric images. Am J Neuroradiol. 10.3174/ajnr.a6848 10.3174/ajnr.A6848 PMC7963227 33154073

[81] Manjón JV, Coupé P (2016) Volbrain: an online MRI brain volumetry system. Front Neuroinform 10. 10.3389/fninf.2016.00030 10.3389/fninf.2016.00030 PMC4961698 27512372

[82] Smith SM, Zhang Y, Jenkinson M (2002). Accurate, robust, and automated longitudinal and cross-sectional brain change analysis. Neuroimage.

[83] Cardoso MJ, Wolz R, Modat M, et al (2012) Geodesic information flows. In: Lecture Notes in Computer Science (including subseries Lecture Notes in Artificial Intelligence and Lecture Notes in Bioinformatics). Springer Verlag, pp 262–270

[84] Prados F, Cardoso MJ, Leung KK (2015). Measuring brain atrophy with a generalized formulation of the boundary shift integral. Neurobiol Aging.

[85] Prados F, Moccia M, Johnson A (2020). Generalised boundary shift integral for longitudinal assessment of spinal cord atrophy. Neuroimage.

[86] Freeborough PA, Fox NC (1997). The boundary shift integral: an accurate and robust measure of cerebral volume changes from registered repeat MRI. IEEE Trans Med Imaging.

[87] Min J, Moon W-J, Jeon JY (2017). Diagnostic efficacy of structural MRI in patients with mild-to-moderate Alzheimer disease: automated volumetric assessment versus visual assessment. Am J Roentgenol.

[88] Heckemann RA, Keihaninejad S, Aljabar P (2010). Improving intersubject image registration using tissue-class information benefits robustness and accuracy of multi-atlas based anatomical segmentation. Neuroimage.

[89] Mettenburg JM, Branstetter BF, Wiley CA (2019). Improved detection of subtle mesial temporal sclerosis: validation of a commercially available software for automated segmentation of hippocampal volume. Am J Neuroradiol.

[90] Koikkalainen J, Rhodius-Meester H, Tolonen A (2016). Differential diagnosis of neurodegenerative diseases using structural MRI data. NeuroImage Clin.

[91] Ross DE, Ochs AL, Seabaugh JM (2013). Man versus machine: comparison of radiologists’ interpretations and NeuroQuant ® volumetric analyses of brain MRIs in patients with traumatic brain injury. J Neuropsychiatry Clin Neurosci.

[92] Azab M, Carone M, Ying SH, Yousem DM (2015). Mesial temporal sclerosis: accuracy of neuroquant versus neuroradiologist. Am J Neuroradiol.

[93] Louis S, Morita-Sherman M, Jones S (2020). Hippocampal sclerosis detection with neuroquant compared with neuroradiologists. Am J Neuroradiol.

[94] Farid N, Girard HM, Kemmotsu N, et al (2012) Temporal lobe epilepsy: quantitative MR volumetry in detection of hippocampal atrophy 1. Radiol n Radiol 264. 10.1148/radiol.12112638/-/DC1 10.1148/radiol.12112638 PMC3401351 22723496

[95] Wang H, Yushkevich PA (2013). Multi-atlas segmentation with joint label fusion and corrective learning—an open source implementation. Front Neuroinform.

[96] Sabuncu MR, Yeo BTT, Van Leemput K (2010). A generative model for image segmentation based on label fusion. IEEE Trans Med Imaging.

[97] Heckemann RA, Hajnal JV, Aljabar P (2006). Automatic anatomical brain MRI segmentation combining label propagation and decision fusion. Neuroimage.

[98] Beadnall HN, Wang C, Van Hecke W, et al (2019) Comparing longitudinal brain atrophy measurement techniques in a real-world multiple sclerosis clinical practice cohort: towards clinical integration? Ther Adv Neurol Disord 12. 10.1177/1756286418823462 10.1177/1756286418823462 PMC6348578 30719080

[99] Vrooman HA, Cocosco CA, van der Lijn F (2007). Multi-spectral brain tissue segmentation using automatically trained k-Nearest-Neighbor classification. Neuroimage.

[100] Granberg T, Uppman M, Hashim F, et al (2016) Clinical feasibility of synthetic MRI in multiple sclerosis: a diagnostic and volumetric validation study. In: Am J Neuroradiol, pp 1023–1029 10.3174/ajnr.A4665 PMC7963550 26797137

[101] Olthof AW, van Ooijen PMA, Rezazade Mehrizi MH (2020). Promises of artificial intelligence in neuroradiology: a systematic technographic review. Neuroradiology.

[102] van Leeuwen KG, Schalekamp S, Rutten MJCM (2021). Artificial intelligence in radiology: 100 commercially available products and their scientific evidence. Eur Radiol.

[103] Rezazade Mehrizi MH, van Ooijen P, Homan M (2021). Applications of artificial intelligence (AI) in diagnostic radiology: a technography study. Eur Radiol.

[104] Scarpazza C, Ha M, Baecker L (2020). Translating research findings into clinical practice: a systematic and critical review of neuroimaging-based clinical tools for brain disorders. Transl Psychiatry.

[105] Raji CA, Ly M, Benzinger TLS (2019). Overview of MR imaging volumetric quantification in neurocognitive disorders. Top Magn Reson Imaging.

[106] Omoumi P, Ducarouge A, Tournier A, et al (2021) To buy or not to buy—evaluating commercial AI solutions in radiology (the ECLAIR guidelines). Eur Radiol 1–11. 10.1007/s00330-020-07684-x 10.1007/s00330-020-07684-x PMC8128726 33666696

[107] Koga H, Yuzuriha T, Yao H (2002). Quantitative MRI findings and cognitive impairment among community dwelling elderly subjects. J Neurol Neurosurg Psychiatry.

[108] Liu CK, Miller BL, Cummings JL (1992). A quantitative MRI study of vascular dementia. Neurology.

[109] Brewer JB (2009). Fully-automated volumetric MRI with normative ranges: translation to clinical practice. Behav Neurol.

[110] Heckemann RA, Hammers A, Rueckert D (2008). Automatic volumetry on MR brain images can support diagnostic decision making. BMC Med Imaging.

[111] Vinke EJ, Huizinga W, Bergtholdt M (2019). Normative brain volumetry derived from different reference populations: impact on single-subject diagnostic assessment in dementia. Neurobiol Aging.

[112] Tolonen A, Rhodius-Meester HFM, Bruun M, et al (2018) Data-driven differential diagnosis of dementia using multiclass disease state index classifier. Front Aging Neurosci. 10.3389/fnagi.2018.00111 10.3389/fnagi.2018.00111 PMC5996907 29922145

[113] Kang KM, Sohn CH, Byun MS (2020). Prediction of amyloid positivity in mild cognitive impairment using fully automated brain segmentation software. Neuropsychiatr Dis Treat.

[114] Klöppel S, Peter J, Ludl A (2015). Applying automated MR-based diagnostic methods to the memory clinic: a prospective study. J Alzheimer’s Dis.

[115] Bron EE, Smits M, van der Flier WM (2015). Standardized evaluation of algorithms for computer-aided diagnosis of dementia based on structural MRI: The CADDementia challenge. Neuroimage.

[116] Goodkin O, Pemberton HG, Vos SB, et al (2020) Clinical evaluation of automated quantitative MRI reports for assessment of hippocampal sclerosis. Eur Radiol 1–11. 10.1007/s00330-020-07075-2 10.1007/s00330-020-07075-2 PMC7755617 32749588

[117] Chagué P, Marro B, Fadili S, et al (2020) Radiological classification of dementia from anatomical MRI assisted by machine learning-derived maps. J Neuroradiol. 10.1016/j.neurad.2020.04.004 10.1016/j.neurad.2020.04.004 32407907

[118] Klöppel S, Stonnington CM, Barnes J (2008). Accuracy of dementia diagnosis - a direct comparison between radiologists and a computerized method. Brain.

[119] Hosny A, Parmar C, Quackenbush J (2018). Artificial intelligence in radiology. Nat Rev Cancer.

[120] Park SH, Han K (2018). Methodologic guide for evaluating clinical performance and effect of artificial intelligence technology for medical diagnosis and prediction. Radiology.

[121] Recht MP, Dewey M, Dreyer K (2020). Integrating artificial intelligence into the clinical practice of radiology: challenges and recommendations. Eur Radiol.

